# The Potential Role of Gut Microbial-Derived Exosomes in Metabolic-Associated Fatty Liver Disease: Implications for Treatment

**DOI:** 10.3389/fimmu.2022.893617

**Published:** 2022-05-11

**Authors:** Binbin Zhang, Jianan Zhao, Minjie Jiang, Dandan Peng, Xiaobing Dou, Yu Song, Junping Shi

**Affiliations:** ^1^ Department of Translational Medicine Platform, The Affiliated Hospital of Hangzhou Normal University, Hangzhou, China; ^2^ Zhejiang University of Traditional Chinese Medicine, Hangzhou, China; ^3^ Guanghua Clinical Medical College, Shanghai University of Traditional Chinese Medicine, Shanghai, China; ^4^ Department of Rheumatology, Shanghai Guanghua Hospital, Shanghai University of Traditional Chinese Medicine, Shanghai, China; ^5^ Institute of Arthritis Research in Integrative Medicine, Shanghai Academy of Traditional Chinese Medicine, Shanghai, China; ^6^ Department of Infectious & Hepatology Diseases, Metabolic Disease Center, The Affiliated Hospital of Hangzhou Normal University, Hangzhou, China

**Keywords:** metabolic-associated fatty liver disease, gut microbial-derived exosomes, therapeutic approaches, insulin resistance, intestinal barrier, inflammatory response, lipid metabolism, liver fibrosis

## Abstract

The prevalence and incidence of metabolic-associated fatty liver disease (MAFLD), a clinically heterogeneous disease whose primary clinical therapies include dietary control and exercise therapy, is increasing worldwide and constitutes a significant medical burden. Gut microbes influence the physiopathological processes of the liver through different mechanisms based on the gut-liver axis. Exosomes are essential carriers of intercellular communication. Most previous studies have focused on adipocyte- and hepatocyte-derived exosomes, while the critical role of microbial-derived exosomes and the molecular mechanisms behind them in MAFLD have received little attention. Therefore, we searched and screened the latest relevant studies in the *PubMeb* database to elucidate the link between microbial-derived exosomes and the pathogenesis of MAFLD, mainly in terms of insulin resistance, intestinal barrier, inflammatory response, lipid metabolism, and liver fibrosis. The aim was to provide a theoretical framework and support for clinical protocols and innovative drug development.

## Introduction

The global prevalence and medical burden of non-alcoholic fatty liver disease (NAFLD), a chronic liver disease that is mainly associated with environmental, dietary, genetic, intestinal, and immune factors, is increasing annually (1). Based on the consensus of expert panels in several countries, NAFLD was renamed as metabolic-associated fatty liver disease (MAFLD). MAFLD is associated with many diseases, including type 2 diabetes, hyperlipidaemia, and obesity; if left untreated, MAFLD can progress to steatohepatitis with liver fibrosis and even liver cancer (2). Its diagnostic criteria include the presence of one of the following three metrics based on hepatic steatosis: overweight/obesity, type 2 diabetes, or metabolic disorders (3–6). The intestine has the largest contact area with the external environment. Its intestinal barrier function is essential for maintaining homeostasis of the organism and includes the intestinal microbial barrier and mucus, gastrointestinal dynamics and secretion, epithelial barrier, immunity (innate and adaptive), and intestinal vascular and hepatic barriers (7). A trillion microorganisms are present in the human gastrointestinal tract. The number of these microorganisms dramatically exceeds the number of human cells. Intestinal microorganisms affect the physiological activities of the host, human metabolism, the immune system, and neurological diseases (8, 9). The intestine and liver may be linked through the portal vein and the transport of enteric-derived products to the liver, thereby affecting bile and associated antibodies in the intestine (10). Gut microbial-derived exosomes can be involved in the physiopathological conditions of the body by transporting a variety of substances. Therefore, in this review, we provide theoretical support for the development of clinical treatment protocols and drugs for MAFLD by discussing the relevance of gut microbial-derived exosomes in the pathogenesis of MAFLD.

## Gut Microflora and MAFLD

Hundreds of millions of intestinal microorganisms live in the gut. These intestinal microbes encode millions of genes, 150 times the number of genes in the human genome, including a rich library of enzymes (11, 12). They provide uncoded enzymes that participate in human metabolism and maintain immune homeostasis through interactions with host cells (13). The gut microbiota is essential for host metabolism and immune homeostasis, and its components contribute significantly to shaping the host immune system (14). The quest to understand the relationship between the host and gut microbiota continues. Dysbiosis of the intestinal flora may lead to various diseases in the host, ultimately compromising health (8, 9). Dysbiosis of intestinal flora is an important pathogenic factor that induces the development and progression of MAFLD (15). The mechanisms by which bacteria influence the progression of metabolism-related diseases are largely unknown, although there is evidence that microbial-derived exosomes are associated with inflammation (16). Typically, bacterial cells communicate with their hosts and other bacteria through direct contact and secretion of soluble products such as metabolites (e.g., short-chain fatty acids), lipopolysaccharides, population-sensing peptides, nucleic acids, proteins, and extracellular vesicles (EVs) (17, 18). For example, butyrate from microbiota can upregulate miRNA-22 expression in hepatocytes, which can decrease Sirtuin1 expression and enhance reactive oxygen species (ROS) production to increase programmed cell death in hepatocytes (19).

## EVs Derived From Gut Microbes

Exosomes were initially thought to be a means of cellular waste disposal until further studies reported their role in mediating intercellular communication, thus attracting significant attention from researchers worldwide (20, 21). Exosomes are a class of EVs with diameters of approximately 30–150 nm (22, 23). Exosomes are derived from most cell types and are present in cell-conditioned media and different biological fluids such as serum, plasma, urine, saliva, ascites, cerebrospinal fluid, and amniotic fluid (24). Enteric bacteria (both pathogenic and commensal) derive EVs for communication with their hosts. These spherical membrane-encapsulated particles transmit some of the biological components of the parental bacteria to the extracellular environment (25). EVs perform different biological functions in the host through different synthetic pathways and mechanisms (26). EVs mainly consist of outer membrane vesicles (OMVs) released by gram-negative bacteria and membrane vesicles (MVs) released by gram-positive bacteria (25). Studies on gram-negative pathogens have shown that OMVs internalize in host cells and promote virulence by delivering cytotoxic factors as well as interfering with immune system mediators (27–29). Currently, microbiota vesicles are considered key players in the signaling process of the intestinal mucosa (25, 30). Gram-negative bacteria follow two major vesicle formation pathways. The first pathway of formation involves blistering of the outer membrane of the bacterial envelope to produce OMVs; the second pathway requires explosive cell lysis to form outer inner membrane vesicles (OIMVs) and explosive outer membrane vesicles (EOMVs) (18). Cytoplasmic membrane vesicles (CMVs) are produced by gram-positive bacteria through endolysin-triggered bulge cell death (26). OMVs are broadly defined as EVs. The blistering membrane process causes OMVs to disrupt the cross-linkage between the outer membrane and the underlying peptidoglycan cell wall layer. In the following sections, we collectively refer to all exosome subtypes as EVs. A variety of functions of exosomes have been characterized, including cell proliferation, differentiation, apoptosis, and immunomodulation (31, 32). Transcriptomic analysis revealed that EVs carry a variety of cargo, including many functional small coding miRNAs, messenger RNA (mRNA), and non-coding RNA (ncRNAs). Proteomic analysis has revealed that EVs also carry other cargo, including membrane-bound proteins, enzymes (e.g. autolysins), toxins, polysaccharides, and peptidoglycans (25, 33). EVs deliver these bioactive components to receptor cells to perform these functions (34).

## Gut Microbial-Derived EVs as an Important Mediator of the Association of Gut Microbes With MAFLD

Gut microbial release EVs that contain specific cargo molecules and have multiple functions (35). Various RNA species (e.g., mRNA, miRNA, tRNA) are among the biologically active components of EVs and may affect host gene expression when delivered to host cells (36–38). In a previous study, the role of OMV in signal transduction between the gut microbiota and the host was demonstrated. OMV produced by the symbiotic probiotic *Escherichia coli* induced the expression and secretion of several cytokines and chemokines in an *in vitro* model (39). EVs contain microRNAs (miRNAs), small coding RNA molecules that regulate gene expression after transcription. miRNAs remain biologically active after delivery to host cells (40). Transfection of Caco2-BBE cells derived from the parental Caco2 strain with mature miR-99b, miR-125a-5p, and miR-1269 decreased the cell growth rate and trans-epithelial resistance, indicating a shift toward the HT29-Cl.19A cell phenotype. EVs may support the transportation process for miRNA to affect cell function (41). Thus, intestinal bacteria use OMV as an essential strategy to communicate with and influence the host responses.

Microbiota-derived EVs are always localised in organs such as the gastrointestinal tract, which is in close contact with bacteria. The biodistribution of EVs derived from the human intestinal commensal bacterium *Bacteroides thetaiotaomicron* after gavage in mice with fluorescent labelling revealed that some EVs accumulated in the liver (42). In addition, by engineering *Escherichia coli* to express Cre recombinase (*E. coli Cre*) colonized into *Rosa26.tdTomato*-background mice, the Cre-LoxP system was used to report bacterial OMV transfer into intestinal epithelial cells and induce fluorescent reporter gene expression, including intestinal stem cells and mucosal immune cells such as macrophages. Outside the intestine, bacterial-derived Cre induces extended marker gene expression in various host tissues, including the heart, liver, kidney, spleen, and brain. *E. coli* OMVs are mainly concentrated in the hepatic confluent region (43). In contrast, endocytosis of endothelial cells is involved in the active transcellular migration of EVs across the epithelial monolayer (e.g., endocytosis, multivesicular body formation, and cytokinesis on the other side of the transcellular layer), which may be a pathway for EVs to cross the intestinal barrier and even the blood-brain barrier (44). Thus, EVs or OMVs released by microorganisms can act as biological shuttle systems for cross-border communication in the host, where bacteria transfer functional biomolecules *via* EVs to individual host cells and target organs, such as the liver. In summary, gut microbes may also be involved in the development of MAFLD through the release of multiple mediators, among which gut microbial-derived EVs may serve as important mediators of the association between gut microbes and MAFLD. Below, we describe the potential effects of gut microbe-derived EVs on various aspects of MAFLD, including insulin resistance, intestinal barrier function, inflammation, lipids, and liver fibrosis.

### Gut Microbial-Derived EVs and Insulin Resistance

Gut microbial-derived EVs influence glucose metabolism by regulating insulin resistance. Insulin resistance is one of the core mechanisms of MAFLD (45). Thus, intestinal barrier dysfunction and increased intestinal permeability in MAFLD, which often implies absorption of host-microbial EVs and lipopolysaccharides (LPS), may lead to a state of insulin resistance (46). Similarly, a study characterising microbial-derived EVs from serum, urine, and stool samples from Korean T2DM subjects found that microbial-derived EVs were prevalent in other parts of the body, such as stool, serum, and urine, whereas gut microbial-derived EVs were less likely to cross the intestinal barrier in healthy individuals (47). It was found that fecal-derived EVs induced insulin resistance and poor glucose tolerance in high-fat diet (HFD)-fed mice compared to conventional diet-fed mice. Macrogenomic analysis revealed that *Pseudomonas panacis (*phylum Proteobacteria*)* EVs can pass through the intestinal barrier to insulin-responsive organs, such as the liver, skeletal muscle, and adipose tissue. They play multiple roles in HFD-fed mice, including blocking insulin signalling pathways in skeletal muscle and adipose tissue, inhibiting insulin-stimulated glucose uptake and GLUT4 translocation in myotubes, and suppressing pAKT expression levels in the skeletal muscle and adipose tissue of mice (48). The skeletal muscle is one of the major tissues involved in peripheral glucose uptake. When free fatty acids are elevated in the body, glucose utilisation by skeletal muscles is inhibited, resulting in insulin resistance (49).

### Intestinal Microbial-Derived EVs and the Intestinal Barrier in Inflammation

As shown above, microbial-driven disruption of the intestinal epithelial and intestinal vascular barriers is a prerequisite for MAFLD, and disruption of the intestinal epithelial and vascular barriers is dependent on the Wnt/β-catenin signalling pathway to promote MAFLD (50). Gene (F11r) encoding junctional adhesion molecule A knockout mice with high fructose, high fat, and cholesterol induce defects in intestinal epithelial permeability, exhibiting more pronounced steatosis (51). The increased intestinal permeability allows the entry of multiple intestinal microbial-derived EVs and LPS, which may be a mechanism contributing to the development of MAFLD. Similar association studies have found increased levels of circulating bacterial EVs in patients with intestinal barrier dysfunction, such as untreated intestinal mucosal inflammation, and levels of bacterial EV-associated LPS correlated with plasma zonulin levels. This suggests that intestinal barrier dysfunction leads to the translocation of bacterial EVs (52). The microbiota affects miRNA expression in the caecum, and their target genes may control the synthesis of proteins related to immune system management and control of intestinal barrier function. Microbial-derived EVs may bridge these gaps and play an important role in maintaining intestinal barrier homeostasis. Microarray analysis of intestinal miRNAs from C57 mice raised under specific pathogen-free (SPF) and germ-free (GF) conditions revealed reduced intestinal miR-10a expression in SPF mice compared to that in GF mice, while GF mice showed decreased intestinal miR-10a expression after decolonization with field bacteria. Intestinal symbiotic bacteria maintain intestinal homeostasis by downregulating dendritic cell miR-10a expression through Toll-like receptor (TLR)-TLR legend (TLRL) interactions and the MyD88-dependent pathway, targeting IL-12/IL-23p40 expression (53). In addition, EVs are considered to have microbial-associated or pathogen-associated molecular patterns (MAMPs) (54). The content of MAMPs in EVs allows them to bind to host pattern recognition receptors (PRRs) in immune and non-immune cells to promote host pathology and immune tolerance, or confer protective immunity (54). *Vibrio cholera* was found to secrete OMV to induce the expression of pro-inflammatory cytokines (e.g., IL-8 and GM-CSF) and chemokines (e.g., CCL2, CCL20, and thymic stromal lymphopoietin) in intestinal epithelial cells in a NOD1-dependent manner through activation of the MAPK/NF-κB pathway. *In vitro* OMV-stimulated epithelial cells can promote the expression of high levels of costimulatory molecules and the release of pro-inflammatory cytokines and chemokines, such as IL-1, IL-6, TNF-α, CCL22, and CCL17, through the activation of dendritic cells (DCs). These cytokines activate CD4+ T cells and promote IL-4, IL-13, and IL-17 release (28). The human commensal bacterium *Bacteroides fragilis* produces a capsular polysaccharide (PSA)-dependent TLR-2 signaling pathway that promotes IL-10 production in dendritic cells (DCs) and prevents chemotactic colitis in mice (55). Thus, microbially derived EVs may be associated with inflammation through multiple immune cells and coordinate the release of multiple biological mediators, particularly MAFLD. For example, gram-negative EVs are characterized by internal phospholipid lobules and external lipopolysaccharide (LPS), a TLR-4 agonist (18). *Escherichia coli* releases OMV containing active pore components of LPS that drive inflammatory responses in human epithelial cells *via* Ca^2+^ signaling and activate TLR-4 (56). In the liver, activation of TLR-4 signaling in hepatocytes, accompanied by nuclear factor kappa B (NF-kB) activation and nuclear translocation, plays an important role in the initiation of MAFLD (57). TLR-4-deficient mice hepatocytes exhibit reduced insulin resistance and obesity-associated inflammation during high-quality diet feeding (58). In addition, systemic deletion of TLR-4 attenuates NASH development in MCD diet-fed mice (59). EVs can also deliver LPS into the cytosol of host cells and activate macrophages *via* TLR-4 receptors (60). In contrast, macrophage activation in MAFLD recruits inflammatory factors and is an essential driver of inflammation. Lipoteichoic acid (LTA) on the surface of gram-positive bacteria and their EVs can bind to TLR-2 to drive the immune response (26). Furthermore, peripheral blood mononuclear cells (PBMCs) stimulated of with *Escherichia coli* strain Nissle 1917 (EcN) or the commensal *E. coli* strain *ECOR12 in vitro* secreted OMV and LPS alone resulting in interleukin (IL)-10, macrophage inflammatory protein 1 alpha (MIP1a), tumor necrosis factor (TNF)-α, IL-6, and IL-8 secretion, implying intestinal inflammation and disruption of the intestinal barrier. Co-culture of intestinal epithelial Caco-2/PBMCs revealed that OMV was internalized by Caco2 cells, thereby preventing the overproduction of multiple pro-inflammatory factors and promoting the production of the anti-inflammatory cytokine IL-10 (39). EcN-derived OMV can promote IL-22 production, transforming growth factor (TGF)-β secretion, and Treg differentiation (39). IL-22 has been shown to enhance intestinal epithelial barrier function (61) and can cooperate with IL-6 to induce the transcription factor *Runx1* to promote the differentiation of Th1 cells to Th17 cells, which promotes inflammation by secreting cytokines such as IL-17, while Treg cells can promote the secretion of the anti-inflammatory factor IL-10 to suppress inflammation (62, 63).

### Gut Microbial-Derived EVs and Adipose Tissue Inflammation

Leaking bacterial products from the gut can also exacerbate the adipose tissue inflammatory response during the progression of MAFLD, leading to insulin resistance and having important effects on systemic metabolism. In addition to metabolites, microbial DNA accumulates in the peripheral circulation and adipose tissues of obese patients (64, 65). Obesity leads to a decrease in the number of complement receptors of the immunoglobulin superfamily (CRIg+) macrophages in human and mouse livers. This phenomenon diminishes the hepatic clearance of circulating bacterial EVs, leading to the spread of bacterial EVs to more distant adipose tissue and inducing inflammation and metabolic disturbances. The intervention of EVs in the intestinal flora of obese mice attenuated the reduction of insulin-stimulated AKT phosphorylation and activation of the cGAS/STING signalling pathway in adipocytes 3T3L-1 cells and enhanced the inflammatory response in adipose tissue (66). Bacterial EVs are enriched in β-cells of obese patients and promote islet inflammatory responses and impair insulin secretion from β-cells by activating the cGAS/STING signalling pathway (67). Similarly, EVs derived from adipocytes of obese patients can promote the release of inflammatory factors such as TNF-α and IL-6 from monocytes and upregulate hepatocyte *TGF-β, PAI-1, MMP-7*, and *TIMP-1* expression to mediate hepatic steatosis and fibrosis (68).

### Gut Microbial-Derived EVs and Liver Fibrosis

Repeated inflammatory stimulation in MAFLD may lead to a poor healing response and, thus, a liver fibrotic response. EVs may be associated with liver fibrosis progression by regulating the expression of miRNAs and their target genes. By assessing miRNA expression profile changes through liver biopsies in patients with no fibrosis or severe liver fibrosis or cirrhosis, 30 upregulated and 45 downregulated miRNAs were found to be present in patients with liver fibrosis (69). In addition, serum levels of IL-6 and miR-233 in patients with NAFLD were correlated. Liver fibrosis was more severe after IL-6 bone marrow-specific knockdown in mice fed a high-fat diet. *In vitro* experiments suggested that the mechanism may involve IL-6 promoting the release of miR-233-containing exosomes from macrophages and reducing the pro-fibrotic gene expression of *TAZ* (70). Similarly, miR-21 regulates fibrosis progression in the liver (71). In addition to regulating miRNA expression, microbial-derived EVs can directly inhibit liver fibrosis by modulating inflammation and maintaining intestinal barrier homeostasis. *Akkermansia muciniphila* primarily inhabits the gastrointestinal mucus layer and is considered a probiotic strain for the treatment of obesity-related diseases (72). Previous studies have shown that heat-inactivated *Akkermansia muciniphila* can modulate the LPS-induced gene expression of liver fibrosis markers, including smooth muscle alpha-actin (α-SMA), tissue inhibitor of metalloproteinase (TIMP), collagen type 1 (Col1), TGF-β, TLR-4, and peroxisome proliferator-activated receptor gamma (PPARγ), in LX-2 cells and reverse the activation of hepatic stellate cell (HSC) in LPS-stimulated LX-2 cells. EVs (50 μg/ml) isolated from *Akkermansia muciniphila* inhibited *TLR-2* and *TLR-4* gene expression in LPS-stimulated LX-2 cells (73). They also reduced the release of serum cytokines TNF-α and IL-6, and increased the level of the anti-inflammatory factor IL-10, thereby suppressing inflammation (73). In addition, *Akkermansia muciniphila* and its EVs increased ZO-1 expression. They inhibited TNF-α and TLR-4 in the colonic tissues of HFD-fed mice, suggesting that they could improve intestinal barrier function and suppress inflammatory responses (73). EVs secreted by *Akkermansia muciniphila* also modulate intestinal integrity and restore disturbed intestinal flora by inhibiting the expression of liver fibrosis markers, including *α-SMA, PDGF, TIMP*, and *Col1a1*, and by suppressing inflammatory genes in experimental mice (73). In addition, MAFLD is associated with renal fibrosis. Live *Akkermansia muciniphila* bacteria and their EV attenuate HFD/CCL4-induced renal tissue injury and the associated gene expression of renal fibrosis, such as α-SMA, PDGF, Col1a1, and TGF-β (73).

### Gut Microbial-Derived EV and Lipid Metabolism

Lipid deposition in MAFLD and lipotoxicity due to oxidative stress are essential drivers of inflammation, and microbial-derived EVs may affect hepatic lipid regulation *via* different mechanisms. *Akkermansia muciniphila* has also been shown to correlate with the lipid content. Changes in *Akkermansia muciniphila* are associated with lipid metabolism in adipose tissue and expression of inflammatory markers and glucose, insulin, triglyceride, and leptin levels in obese mouse models (74). A similar study found that post-pasteurization *Akkermansia muciniphila* can affect intestinal lipid absorption and metabolism, thus regulating body weight and fat gain by regulating perilipin2 lipid droplet-associated factors (75). In addition, gavage of *A. muciniphila I (Amuc_GP01)* in an HFD-induced experimental mouse model improved glucose tolerance, hyperlipidemia, and hepatic steatosis (76). Furthermore, in the HFD-induced intestinal microbiota of mice, triglyceride content in the liver was positively correlated with miR-21 expression, whereas hepatic miR-21 expression was positively correlated with *Firmicutes* and negatively correlated with *Proteobacteria* and *Bacteroides acidifaciens*. The ability of the bacterial antigens to regulate hepatic miRNAs was tested *in vitro*. The pro-inflammatory LPS of *E. coli O55:B5* promoted miR-21 expression in primary wild-type mouse hepatocytes in a dose-dependent manner, suggesting that gut microbes, possibly through their antigens (e.g., LPS), regulate hepatic miRNA expression to influence the liver (77). The regulatory effects of miRNAs on gut bacteria and the liver need to be further validated by *in vivo* experiments. In aged DB/DB mice, long-term (18 weeks) inhibition of miR-21 reduces body weight and adipocyte size (78). In addition, it was found that intestinal flora disorders in MAFLD abnormally increased the *Bacteroidetes* ratio and *Streptomyces* spp., and that high mobility group box 1 (HMGB1) promoted hepatic steatosis through transfer from the intestine to the liver (79).

In addition to the potential impact of microbial-derived EVs on lipid metabolism in MAFLD, most studies have found relevance of other source-derived exosome secretions to MAFLD, particularly lipid-regulated EVs release. For example, plasma exosome levels are significantly elevated in MAFLD patients. Fatty acid-induced damage-regulated autophagy modulator (DRAM) enhances hepatocyte-associated exosome release by promoting lysosomal localization of stomatin upon interaction with stomatin, which may serve as a potential biomarker of MAFLD (80). The presence of many membrane-bound vesicles generated by hepatocytes in experimental mice with steatohepatitis that respond to FFA-derived EVs correlates significantly with disease severity (81). Similarly, adipocytes can endocytose HepG2 cell-derived exosomes, thereby promoting their inflammatory phenotypic differentiation by activating phosphokinase and NF-kB signalling and recruiting inflammatory macrophages to co-promote the inflammatory milieu (82). The most relevant research advances have been reviewed widely (83–86). Without going into much detail, we note that the potential relevance of excessive lipid deposition in MAFLD and its regulation in microbially derived EVs deserves further investigation.

## Future Perspective and Challenges

Many cited studies still use the term “NAFLD” and link to other metabolic diseases such as T2M. In view of the update of NAFLD terminology and diagnostic criteria to further standardise and advance clinical and research progress in the discipline of hepatology, we use the term “MAFLD” instead of “NAFLD” throughout the text. We believe that it is extremely important to standardise and update this terminology; however, the diagnostic distinction between them should be noted in the future. Sumida et al. summarised the current pharmacological treatments for MAFLD and the possible challenges (87). In summary, there are no FDA-approved treatment options for MAFLD, and diet control and exercise remain important lifestyle interventions. In addition, several drugs have potential therapeutic effects by targeting different mechanisms, such as lipid deposition (elafibranor), oxidative stress, inflammation, cell death (emricasan), intestinal microenvironment and metabolism (IMMe124), and antifibrotic drugs (cenicriviroc), some of which are already in clinical trials (87). As mentioned above, gut microbial-derived EVs might affect MAFLD through different mechanisms; therefore, the proposed increase in beneficial gut microbes (flora transplantation, genetic engineering) or reduction of harmful gut microbial populations (lifestyle changes, pharmacological interventions) are potentially important approaches. Probiotics, prebiotics (fermented products that improve the flora composition of patients) (88) and prebiotics (combination of probiotics and dietary or food components) can improve the extent of experimental animal models and MAFLD patients. For example, the intake of *Lactobacillus acidophilus La5 and Bifidobacterium lactis Bb12* improved liver enzymes, total serum cholesterol, and LDL cholesterol levels in patients with MAFLD (89). Prebiotics can significantly reduce TNF-α, CRP, liver enzymes, and steatosis in patients (90). Most of the current studies on MAFLD and gut flora have focused on changes in the abnormal gut flora and the influence of the flora on a particular aspect of MAFLD and have not directly focused on the role of gut microbial-derived EVs. This is an important mechanism by which the intestinal flora exerts its physiopathological functions. Considering the preliminary studies on the potential benefits of probiotic transplants, prebiotics, and prebiotics for MAFLD treatment in combination with the role of EVs in MAFLD, we believe this will be a direction with great research potential in the future; however, further well-designed preclinical and clinical trials are still needed for in-depth studies.

## Discussion

**Figure 1:**
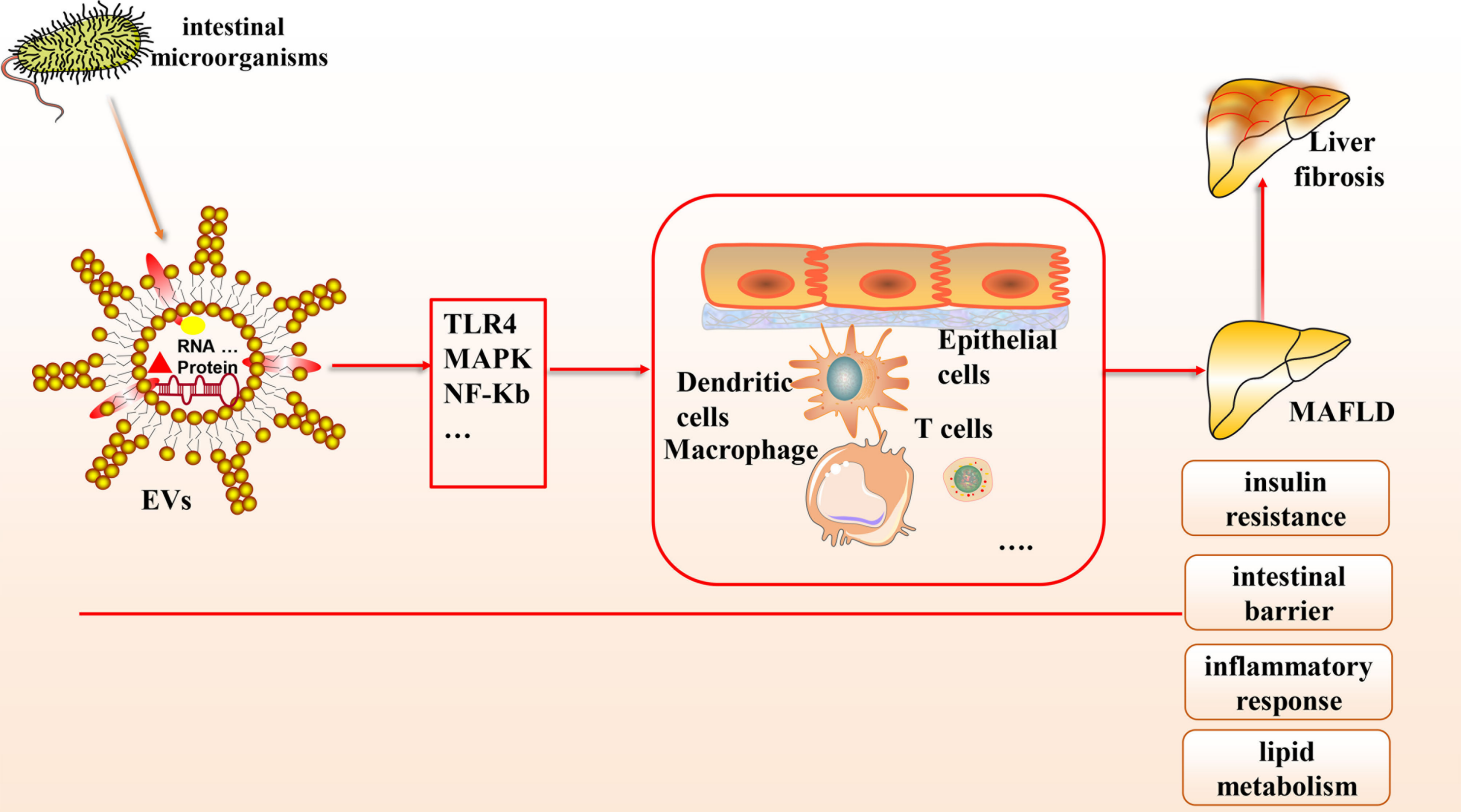
The connection between gut microbial-derived exosomes and MAFLD. Many microorganisms exist in the human body, which can secrete EVs, a bilayer lipid membrane structure that can include the outermost LPS, cytoplasmic and membrane-bound proteins, DNA, multiple protein components, etc. Based on the gut-liver axis theory, EVs can carry various biological mediators and affect multiple cells through many signaling pathways to influence pathological mechanisms in MAFLD, including intestinal barrier homeostasis, insulin resistance, lipid metabolism, inflammatory response, etc.

Based on the gut-liver axis theory, gut microbes are an essential component of the human body, and their derived EVs, which contain abundant microbial DNA, proteins, and lipids, have been gradually recognized to contribute to the pathological mechanisms of various liver diseases, especially MAFLD (see **Figure 1**). In this article, we provide a theoretical basis for developing innovative clinical treatment options for MAFLD based on gut microbial-derived EVs by discussing their potential relationship with insulin resistance, the intestinal barrier, inflammation, lipid metabolism, and liver fibrosis and the improvement of MAFLD through probiotic colonization. There is great potential to elucidate the molecular composition of the cargoes carried by EVs and to explore how they interact with receptor cells and their mechanisms by combining a variety of modern molecular biology techniques and multi-omics applications.

## Author Contributions

BZ and JZ are responsible for the collection, collation and writing of the original manuscript. MJ, and DP are responsible for the collection of the original manuscript. XD, YS, and JS are responsible for the revision and review of the manuscript. All authors reviewed and accepted with the final version.

## Funding

This work was funded by the National Natural Science Funds of China (81570524), Zhejiang Provincial Basic Public Welfare Research Project (GF20H030035), Major Projects of Hangzhou Medical and Health Science and Technology Program (0020191059).

## Conflict of Interest

The authors declare that the research was conducted in the absence of any commercial or financial relationships that could be construed as a potential conflict of interest.

## Publisher’s Note

All claims expressed in this article are solely those of the authors and do not necessarily represent those of their affiliated organizations, or those of the publisher, the editors and the reviewers. Any product that may be evaluated in this article, or claim that may be made by its manufacturer, is not guaranteed or endorsed by the publisher.
